# Hemodynamics Challenges for the Navigation of Medical Microbots for the Treatment of CVDs

**DOI:** 10.3390/ma14237402

**Published:** 2021-12-02

**Authors:** Erica Doutel, Francisco J. Galindo-Rosales, Laura Campo-Deaño

**Affiliations:** 1 CEFT, Departamento de Engenharia Mecânica, Faculdade de Engenharia da Universidade do Porto, 4200-465 Porto, Portugal; erdoutel@fe.up.pt; 2 CEFT, Departamento de Engenharia Química, Faculdade de Engenharia da Universidade do Porto, 4200-465 Porto, Portugal; galindo@fe.up.pt

**Keywords:** microbots, complex fluid flows, hemodynamics, pulsatile flow

## Abstract

Microbots have been considered powerful tools in minimally invasive medicine. In the last few years, the topic has been highly studied by researchers across the globe to further develop the capabilities of microbots in medicine. One of many applications of these devices is performing surgical procedures inside the human circulatory system. It is expected that these microdevices traveling along the microvascular system can remove clots, deliver drugs, or even look for specific cells or regions to diagnose and treat. Although many studies have been published about this subject, the experimental influence of microbot morphology in hemodynamics of specific sites of the human circulatory system is yet to be explored. There are numerical studies already considering some of human physiological conditions, however, experimental validation is vital and demands further investigations. The roles of specific hemodynamic variables, the non-Newtonian behavior of blood and its particulate nature at small scales, the flow disturbances caused by the heart cycle, and the anatomy of certain arteries (i.e., bifurcations and tortuosity of vessels of some regions) in the determination of the dynamic performance of microbots are of paramount importance. This paper presents a critical analysis of the state-of-the-art literature related to pulsatile blood flow around microbots.

## 1. Introduction

Cardiovascular diseases (CVDs) are the leading cause of death worldwide (17.9 million) [1]. In Europe, half of all deaths are from CVDs [2]. The number of hospitalizations, rehabilitation services, pharmacological and technological treatments, invasive surgical procedures, and recovery time make CVDs a burden on health systems [3]. This opens a wide field to search for minimally invasive diagnosis and treatments for cardiovascular diseases. Emerging exploratory research in microtechnology encompasses the development of tiny robots able to access inaccessible regions in circulatory systems as well as to substitute some expensive and critically invasive surgical procedures that could be a starting point in minimizing the recovery time. Thus, microbots are microelectromechanical devices (MEMs) or small devices made of multifunctional smart materials, structures, and mechanisms that can navigate controllably by means of an untethered manipulation system and have access to small and constrained locations or workspaces to perform simple tasks, such as pushing or carrying a cargo [4,5]. Martínez-Aranda et al. [6] characterized the complex fluid flow dynamics around individual microbots. They studied the influence of different microbot shapes on the flow dynamics using a novel microfluidic hydrotunnel. Additionally, they analyzed in this microchannel the Newtonian and non-Newtonian rheological flow behavior around simplified 3D microbot prototypes using μ-PIV measurements. They extracted an estimation of the energy dissipated in the flow by the microbots’ presence in the center of the channel. Nevertheless, the flow disturbance caused by either oscillating flow or tortuosity of the vessel was not explored and, to the best of the authors’ knowledge, this area of knowledge still remains uncharted. Later on, Campo-Deano [7] described the implications of different experimental techniques in microfluidics such as flow visualizations, pressure drop measurements, μ-PIV, and birefringence analysis, to assess the most efficient shape and design for a microbot using a blood analogue with non-Newtonian rheological behavior.

Other methodologies concerning numerical methods such as computational fluid dynamics (CFD) have been used to analyze flow behavior regarding hemodynamic analysis in blood vessels to better understand the development of CVDs [8,9,10]. There are some computational studies of flow surrounding 3D microbots inside channels mimicking the vascular system to understand how the flow field is altered with the introduction of a microbot inside fluid-filled channels. Temel and Yesilyurt [11] presented a work based on numerical simulations of the flow field to analyze the motion by the interaction of the microbot with the channel wall. With recent advances in computational technology, more investigations using computational assessment tools to clarify microswimmer motion in order to find the most suitable design have emerged. Acemoglu and Yesilyurt [12] analyzed by a 3D CFD model the swimming of a microorganism with a single helical flagellum in circular channels. They analyzed the effect of some geometric parameters and validated them with experimental results reported in the literature, obtaining an optimal shape for the speed and the power efficiency. Later on, Caldag and Yesilyurt [13] proposed a kinematic model that uses CFD for the simulation of a helical microswimmer inside cylindrical channels to investigate the flow dynamics under confinement. Due to the complexity of the blood vessel wall structure, once they are not rigid and mostly are defined as a hyperplastic material [14], the necessity to analyze the interaction of blood flow and vessel walls is of paramount importance. However, most of the studies for simplification do not take into account the relation and reaction of fluid–structure interaction (FSI) (blood flow–wall of vessel). To comprehend the differences and the impact of the blood vessels’ dynamic behavior [15,16], the CFD-FSI methodology has recently enhanced some investigations in the field. Numerical studies involving the analysis of blood flow surrounding a 3D microbot should couple both CFD and FSI methods. This is of utmost importance since some blood vessels could suffer deformation during the cardiac cycle [17].

Despite the recent advances in implementing experimental and numerical studies related to the hemodynamics around microbots, these investigations are still scarce. Understanding the influence of the microbots’ morphology on the flow dynamics to have an efficient device capable of moving through different blood vessels, especially to minimize CVDs, considering the vessels’ morphology, non-Newtonian properties of blood, and pulsatile flow, is of paramount importance and needs more attention.

In this review, the most important potential applications of the use of microbots in the human circulatory system are summarized in Section 2. The most relevant microbots shape-designed for the circulatory system are described in Section 3. In Section 4, the human circulatory challenges are described, with a detailed description of the cardiac cycle, the blood vessel network, and the most characteristic dimensionless numbers of the hemodynamics. Section 5 describes the importance of considering the pulsatile flow in experimental and numerical approaches and how to perform them.

## 2. Applications of Microbots in the Human Circulatory System

**Figure 1:**
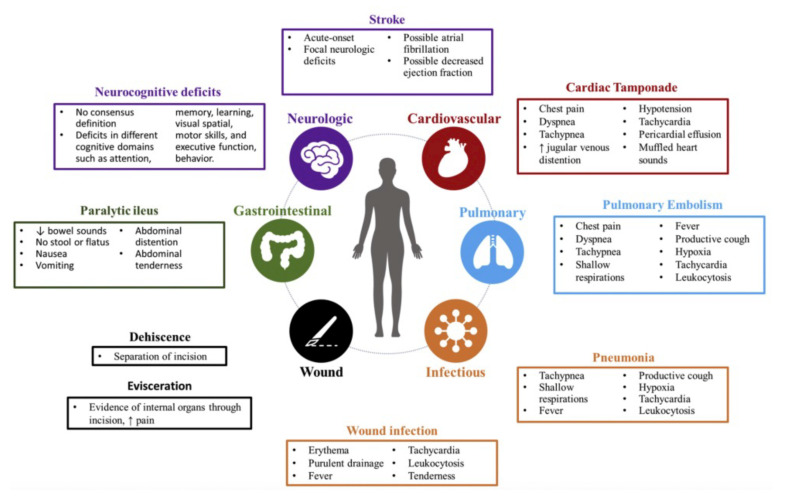
Common complications following CABG surgery, with associated signs, symptoms, and laboratory findings. Reprinted from the American Journal of Emergency Medicine, 36(12), Montrief, T., Koyfman, A., and Long, B. Coronary artery bypass graft surgery complications: A review for emergency clinicians, 2289–2297 (2018), with permission from Elsevier [21].

CVDs are the leading cause of death worldwide; more people die annually from CVDs than from any other cause. Among cardiovascular disorders, the major causes of death are due to vascular obstructions, caused mainly by thrombus formation or atherosclerotic plaques [1,18]. The formation of atherosclerotic plaques in arterial walls reduces their diameter, causes a limitation in blood supply to vital organs, and could lead to a blocked clot causing fatal accidents. The circulatory system is responsible for transporting oxygen and nutrients to the whole body. A failure such as an obstruction in this vascular system could lead to a heart attack, stroke, critical limb ischemia, or in veins could even lead to a deep thrombosis and pulmonary embolism [19]. The therapeutic solutions currently used to treat CVDs are many and include several strategies. Depending on the severity of the cardiovascular disease and the location in the vascular system, the surgical procedure is then chosen. For example, coronary arteries are responsible for the blood supply of the heart muscle, and an occlusion in that specific location represents an acute disease. One of the most invasive procedures is the coronary artery bypass graft (CABG) [20]. During this surgery, the chest bone is opened to access the heart. Medications are given to stop the heart and a heart–lung bypass machine is used to avoid circulation in the blocked vessel and to keep blood and oxygen moving through the entire body. After surgery, blood flow to the heart is restored. There are other options to avoid making a large incision to open the chest bone such as the minimally invasive direct coronary artery bypass grafting where several small incisions are made on the left side of the chest between the ribs. This type of surgery is used for bypassing the blood vessels in front of the heart. CABG is well known for its limitations such as high risk of surgical mortality, high cost, and long recovery time [20]. A more detailed illustration of the several complications involved in this type of medical intervention is shown in Figure 1.

Alternative procedures are preferred whenever possible, such as percutaneous coronary intervention (PCI). It is a reliable procedure that is much less invasive and expensive than CABG, and it combines a coronary angioplasty with stenting [22,23]. PCI consists in destroying the clogged blood vessel by making a hole and enlarging it using a balloon or a drug-eluting stent. Typical complications include detachment of fragments, vessel damage, elastic recoil, restenosis, and infection [22]. Intravascular catheters equipped with MHz-frequency ultrasound transducers for enhancing dissolution of thrombi have been developed by Parikh et al.(16) and ultrasound is proven to significantly accelerate the action of thrombolytic drugs, reducing the treatment time, which may be of value in improving stroke therapy. However, the collateral effect of ultrasound exposure is cavitation which leads to problems of dosimetry and control. The role of this method and the full range of its clinical usefulness for thrombolysis are still being evaluated and need to be better explored [24]. Miloro et al. [23] enumerated and described therapeutic strategies currently used to remove vascular obstructions, which present an alternative path to traditional angioplasty and the stenting process. In their review, they classified and divided the therapeutic strategies into mechanical, laser, chemical, and hybrid removal strategies concerning their working principles, and highlighted the physical mechanisms behind each solution, illustrating some tools, devices, and procedures currently considered as minimally invasive techniques for removal procedures of thrombi and atheroma.

**Figure 2:**
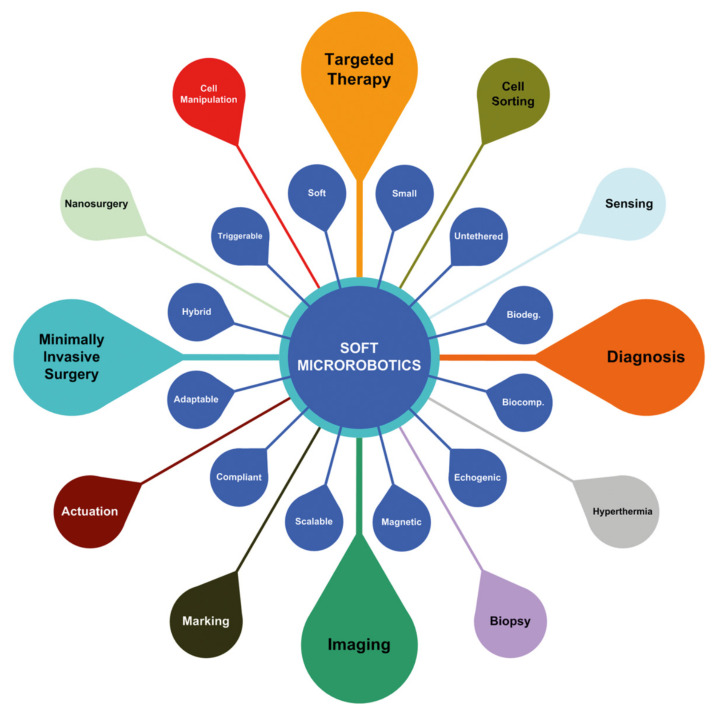
Characteristics and application areas of soft microbots. The unique features of microbots make them suitable for a variety of biomedical applications. Reprinted from Mathematical Modeling of Swimming Soft Microrobots, Islam S.M. Khalil, Anke Klingner, Sarthak Misra, Chapter 1—Introduction: Soft Microrobots, 1–15, 2021, with permission from Elsevier [5].

Minimally invasive procedures underwent significant advances in the last decade. A good example of this is the use of tiny robots for operations in the vascular network. Microbots offer significant opportunities in future targeted medical interventions (Figure 2). They have the potential to perform tasks that are currently difficult or impossible with the techniques used currently. If these microbots are driven in the blood circulatory system, a very large number of remote locations in the human body could become accessible. Looking to the future, Miloro’s classification is a good starting point to be aware of the recent advances regarding minimally invasive interventions in the circulatory system.

According to Miloro’s classification [23], one therapeutic strategy in the vascular system would be mechanical removal. It combines mechanical or fluid-mediated actions (i.e., milling, grinding, maceration, dragging, scraping, suction, shear, and ultrasounds) between a milli/microdevice and the arterial obstruction [23]. Several types of thrombectomy/atherectomy as well as ultrasound thrombolysis and ultrasound angioplasty were extensively analyzed concerning their physical implications in removing vascular obstructions. Regarding the minimal invasiveness, easy use, and low-cost implications of both thrombectomy and atherectomy, there are some clinical complications depending on the devices/tools or actions that are involved. The most common are endothelial denudation, vessel perforation/dissection, obstruction of small vessels due to the larger fragments of thrombi causing a distal embolization, cutter size constraints with the inability to remove thrombi/atheroma, and hemodynamic failures.

Another interesting strategy proposed in Miloro’s classification [23] would be laser removal, which covers all the therapies that involve photochemical, photothermal, and photomechanical actions to remove vascular obstructions. Some limitations are described regarding laser–material interaction within the vascular system as well as the use of ultrasound therapies. Laser therapies by means of ultraviolet (UV) lights using photochemical actions have an advantage compared to laser therapies using a photothermal process. The UV light process relies on a short penetration depth and aims to break molecular bonds directly by a lytic agent, ablating a thrombus and inhibiting platelet aggregation. On the other hand, in the thermal process, the laser energy has direct contact with the obstruction surface, and it is dynamically worn down [25]. Temperatures higher than 43–45 °C could lead to retraction, shrinkage, coagulation, and carbonization [26]. Therefore, in the context of the applicability, thermal actions in the vascular system are purposively avoided and need to be better explored, particularly to remove vascular obstructions.

It is important to note that microbots could be well suited to perform tasks by means of mechanical, laser, and hybrid removal actions. They could be adapted with specialized design tools to accomplish those tasks, or they can act as simple static structures whose positions are handy or even self-oriented. They could be easily used in a rotary motion for scraping a fatty material from the internal wall of blood vessels or use a resonating mechanical structure to emit ultrasonic pressure waves to destroy a calcified material which could be obstructing a vessel [26,27,28]. As a static structure, the microbot could itself act like a drug-delivered stent and navigate and be deployed in the region of interest to keep passageways open for blood to keep flowing through a clogged vessel. Another functionality that could be interesting as a static structure is a microbot acting like an occlusion itself to clog a blood vessel to intentionally starve a region of nutrition [27]. They could even be programmed for administrating therapy for aneurysms by remote control or even by wireless transmission.

Another approach would include chemical removal strategies involving chemical reactions such as chemical dissolution or disruption of a lytic agent to target and destroy the biological structure of obstructions. These types of clinical therapies (i.e., intravenous thrombolysis, intra-arterial thrombolysis), besides easy administration, widespread availability, and proven efficacy, only attack thrombi in arteries and veins and could lead to some clinical risks such as intracranial hemorrhage, systemic hemorrhage, immunologic complications, hypotension, and myocardial rupture [29]. Several authors refer to the use of microbots in targeted therapy [26,27,28]. Microbots could perform targeted therapy as they can deliver the chosen drug, increasing its concentration in a specific region while reducing the side effects in the rest of the body [27,28]. Dogangil et al. [29] developed a microbot for targeted retinal drug delivery to destroy a common obstruction in blood flow of the retina vascular system, caused by clot formation.

Dogangil et al. [29] concluded that the use of microbots controlled by a magnetic field in targeted drug delivery is feasible and possible to accomplish; however, it should be better explored for clinical efficacy. Indeed, targeted drug delivery requires advanced coating techniques to load the drugs into microbots and the drug delivery kinetics need to be better controlled.

Finally, another potential strategy suggested by Miloro et al. [23] focuses on the interaction between both mechanical and chemical actions described above.

## 3. Microbots Designed for the Circulatory System

Microbotics research has recently become an important topic regarding the design, study, and application of miniature robots for medical and biological purposes. These robots are generally mobile robots or *microswimmers* with characteristic dimensions less than 1 mm. Recently, there have been many applications and several novel designs of microbots that have emerged; nevertheless, before introducing them, it is necessary to give a simple overview in order to specify the type of microbot that we are describing in this review. Most of the promising microswimmers are designed to make delicate micromanipulations in unreachable environments for medical purposes. Those environments inside the human body are conditioned by all the body fluids, and it is expected that these tiny robots will swim in these fluid environments, traveling and applying their functionality in different parts of the human body. The main environment that we are focused on in this review is the circulatory system, but there are other locations in the human body where microbotics research has been successfully applied, such as the gastrointestinal system [30], urinary system and prostate [31], central nervous system [32], eye [29], and cardiovascular system [33,34]. There are multiple types of microbots and several classifications. Campo-Deaño [7] systematized the microbot classification based on their fabrication and/or propulsion method. According to this classification, there are five major types of microbots: electrostatic, magnetic, optically actuated, catalytic, and biohybrid microbots.

**Figure 3:**
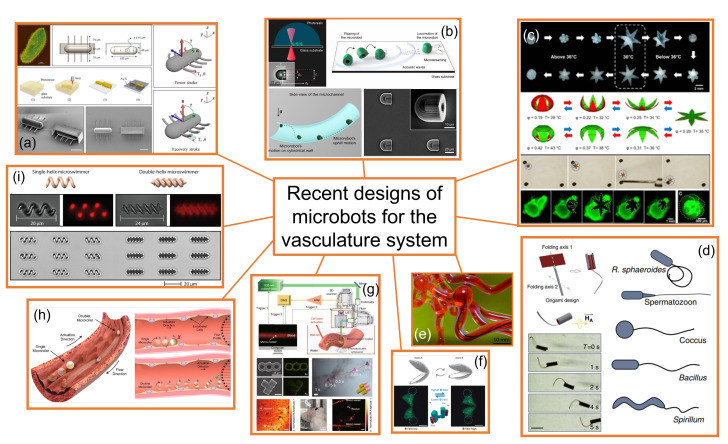
Some of the most recent microbots designed for the vasculature system: (**a**) ciliary microbot [35]; (**b**) soft attractor wall microbot [36]; (**c**) self-folding microbot [38]; (**d**) sperm-shaped microbot [40]; (**e**) snake-shaped microbot [41]; (**f**) scallop-shaped microrobot [42]; (**g**) microrocket robot [43]; (**h**) surface microrollers [45]; (**i**) helical microbots [46]. All images have been reproduced with permission from the corresponding publishers, therefore further requests for permission related to the material excerpted should be directed to them.

In this review, we are focused on the shape of the microbots, as it is responsible for allowing the best hemodynamic performance of a microswimmer inside the circulatory system. To reach the best design for proper locomotion through the vasculature system, several researchers around the globe are developing different shapes for microbots. In this sense, it is worth mentioning the worldwide leading position of Sitti and co-workers of the Physical Intelligence Department at the Max Planck Institute for Intelligent Systems in the development of pioneering designs of miniature medical mobile robots [4]. Figure 3 summarizes some of the most recent and relevant microbots specifically designed to work in the bloodstream:

(a) **Ciliary microbots**. Kim et al. [35] developed and fabricated a ciliary microbot, allowing the cilia to beat back and forth using an electromagnetic coil system, providing the possibility of moving at a speed of about 340 micrometers per second, with a much greater range of maneuvrability than previous microbots operating under magnetic attraction drive, as the controller can shift the angle of the microbot from zero to 120 degrees. Thus, ciliary microbot locomotion represents a high-efficiency swimming method in viscous fluid with non-reciprocal actuation; however, the 220 micrometer-long robot would limit the range of vessels. This kind of microbot may be useful for drug delivery purposes.
(b) **Soft attractor wall microbot.** Aghakhani et al. [36,37] developed a 3D bullet-shaped microbot containing a spherical air bubble in its internal cavity where the bubble resonates using acoustic waves. They combined the acoustic powering with magnetic steering with the purpose of effective microbot actuation and navigation in confined and hard-to-reach body locations. This design would allow targeted drug delivery. Swimming near the vessel walls is an efficient type of propulsion as high-speed blood flow is avoided; however, lossy material, such as body tissues, would require generating sufficient acoustic radiation force, and surface microtopography of blood vessels, in the size scale of the microbots, would represent a major hurdle for robust locomotion of surface-rolling microbots against the blood flow.
(c) **Self-folding microbot.** Breger et al. [38] proposed a self-folding functional microbot gripper with embedded iron oxide (Fe_2_O_3_) nanoparticles in the porous hydrogel layer, allowing the microgrippers to be responsive and remotely guided using magnetic fields. They illustrated the operation and functionality of these polymeric microgrippers for soft robotic and surgical applications, such as capturing and performing an excision of cells, such as fibroblast clumps. They could also be used for drug delivery purposes. Small oscillations in the temperature of blood would modify the shape of the microbot and, therefore, may alter its hemodynamics without control.
(d) **Sperm-shaped microbot.** Another microbot inspired by natural structures consists of a head connected to a flexible tail, similar to a sperm cell [39,40]. This kind of microbot uses weak oscillating magnetic fields for steering and propulsion. Organisms that move around in a biological system use non-reciprocating devices, such as flagella or cilia, and this has been naturally proven as an efficient way of swimming in non-Newtonian fluids. It could be used for drug delivery and clearing out clogged arteries. Being soft, flexible, and motorless is positive for its application in the cardiovascular system.
(e) **Snake-shaped microbot** [41]. Kim et al [41], introduced another class of magnetically actuated soft robots. They are based on ferromagnetic soft materials and magnetic polarities were programmed in the body of the robot, which confers the advantage of enabling navigation through complex, narrow, fragile, and tortuous environments and moving around the structure of veins in the brain. These devices may also be used for light transportation, enabling the possibility of performing a photoablation treatment of clots. Thus, they could be used for the diagnosis and treatment of blood clots and aneurysms, as well as performing other small-scale operations in the brain. Despite a hydrogel slime cover minimizing friction during travel, the increasing contact with the solid surface of the microbot may damage deteriorated vessels walls.
(f) **Scallop-shaped microbot.** This design was proposed by Qiu et al. [42] and is able to paddle through non-Newtonian fluids such as blood and plasma, but also in other biofluids. It is striking that this design uses a reciprocal method of movement to propel the microscallops, as this generally does not work in such fluids. This device would be useful for drug delivery purposes. Its simple design allows for 3D printing fabrication; however, its 800-micron size would limit the size of the vessels through which it could swim.
(g) **Microrocket robot**. This design was recently proposed by Li et al. [43] and solved two of the major challenges in small mobile robots in the biomedical field, that is, driving at high speed through a viscous media and observing the position of a single microbot inside the bloodstream. A moving speed of 2.8 mm/s under near-infrared light actuation in a static Newtonian analogue solution was reported; however, its performance through real blood may be different.
(h) **Surface microrollers.** Alapan et al. [44] reported cell-sized multifunctional surface microrollers with 3.0- and 7.8-micrometer diameters for targeted drug delivery into specific cells and controlled navigation. The spherical microrollers consist of magnetically responsive Janus microparticles that can be functionalized with specific targeting antibodies against cancer cells and or light-cleavable cancer drug molecules. Swimming near the vessel walls is an efficient type of propulsion as high-speed blood flow is avoided, thus the microrollers can be propelled against physiologically relevant blood flow in endothelialized microchannels and can even move upstream on inclined three-dimensional surfaces in physiologically relevant blood flow. Surface microtopography of blood vessels, in the size scale of the microbots, is a major hurdle for robust locomotion of surface-rolling microbots against the blood flow.
(i) **Helical microbots**. Helical microbots are another commonly studied design, aiming at targeted drug delivery, cell sorting, and cell characterization. They used helical propulsion to break time-reversal symmetry and provide locomotion at a low Reynolds number in the bloodstream via the action of a uniform or non-uniform rotating magnetic field. Helical microbots could be used for rubbing against blood clots [39]. Khalil et al. [39] experimentally investigated the influence of lysis and rubbing on the removal of clots at a low Reynolds number inside catheter segments, which was performed by helical robots. They also developed a model to predict an optimal rubbing frequency of the helical robot to reach a maximum removal rate, showing better mechanical grinding of blood clots than chemical lysis. Nevertheless, the influence of the tip geometry on the removal rate on the 3D network of blood clots will need to be tested. The size and lack of flexibility may damage the walls of the vessels and limit the size of the vessels in applications.

Of course, all the above-mentioned designs would require further analysis of the biocompatibility and long-term tests to assess the toxicity in in vivo/in vitro environments before application in real cases. Concerning biocompatibility, safety risks could be mitigated by constructing the microbots from materials intrinsically compatible with physiological environments. Ceylan et al. [46] proposed a personalized approach by using patient blood–derivable biomaterials as the main construction fabric of wireless medical micromachines to alleviate safety risks from biocompatibility. They demonstrated that it is possible to 3D print multiresponsive microswimmers and microrollers made from magnetic nanocomposites of blood plasma, serum albumin protein, and platelet lysate. Moreover, their degradability with proteinases lowers the risks of long-term toxicity. This personalized micromachine fabrication strategy seems to be the key for future medical robots and devices to improve biocompatibility and smart functionality.

Table 1 lists Miloro’s classification [23] of the strategies for removal procedures of thrombi and atheroma, and in which of those the above-described new designs of microbots could be useful in each therapeutic strategy.

Thus, although in recent years there has been a huge advance in microbot technology, particularly in the design of microbots dedicated to the treatment of cardiovascular diseases and drug delivery purposes through the human circulatory system, there is still much to be done in this multidisciplinary field, particularly in the analysis and study of the hemodynamics of the microbots in the human circulatory system, which would allow understanding and determining which are the physical parameters of the physiology that could offer potential challenges for microbot applicability in the treatment and diagnosis of CVDs.

## 4. Human Circulatory Challenges

**Figure 4:**
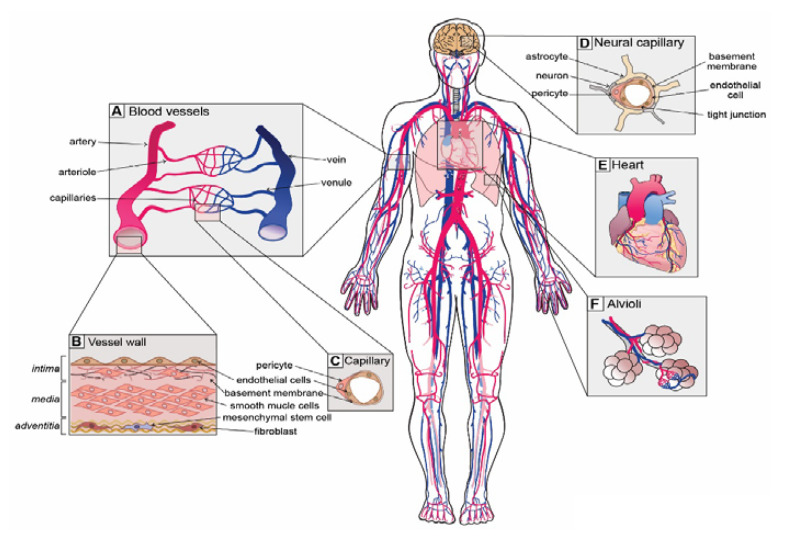
Overview of circulatory system composed of the heart as a pump, fluid in red (oxygen-rich blood) and in blue (oxygen-poor blood), and the network of different blood vessels (in red—arteries and in blue—veins). Reproduced with permission from [50].

From the engineering point of view, the human circulatory system could be explained as a fully optimized in vivo fluid mechanics lab that works in perfect harmony. This living lab is equipped with a closed circuit composed of several channels with different calibers (blood vessel network), a brilliant and well-designed pump (the heart), and a complex fluid with incomparable properties (blood). When we imagine a micro/nanorobot swimming through this system it is easier to compare this lab to a science fiction movie, as Sitti et al. [47] once wrote. To better understand this huge environment (the circulatory system), it is important to explain how it works. Starting from the heart left ventricle, the blood is pumped to the systemic circulation through the aorta and flows through a pipeline network of large arteries and is subdivided into smaller arteries until microcirculation. Here, the blood vessels continue their subdivision into arterioles and finally into capillaries where the exchange of nutrients and waste bioproducts is vital for the living tissues. Then, capillaries merge into venules, and then join into veins and flow towards the right side of the heart where blood is again pumped to enter in pulmonary circulation where gas exchange occurs, and then it comes back to the left side of the heart via the pulmonary vein [48,49]. A schematic diagram of this system is shown in Figure 4. It is foreseen that microbots could navigate through this very complex and huge system, which is why throughout this review we will point out which are the main hemodynamic challenges regarding their performance.

### 4.1. Blood Vessel Network

The blood vessels are absolutely essential in this system to ensure the normal distribution of blood containing nutrients, oxygen, water, and hormones to all living tissues and organs as well as to remove the metabolic waste byproducts and carbon dioxide from all the tissues and organs on the way to the lungs. They are essential to blood pressure regulation and to the maintenance of body temperature.

#### 4.1.1. Type and Function of Blood Vessels

The blood vessels can be classified into five types, i.e., arteries, arterioles, capillaries, venules, and veins; however, we could simplify and classify them into three major groups by their function and structure:

**Group I: Arteries and small branches (arterioles)**: the main function is to distribute from the heart the oxygen-rich blood with all metabolic byproducts to all regions of the body. These vessels are normally highly pressurized, excluding the pulmonary artery. Their structure is composed of three layers called the tunica intima, media, and adventitia. They can be divided into three types by the structure of their walls and main functions:

- Elastic arteries (i.e., aorta and pulmonary arteries), which are characterized by a thin wall and a larger diameter (normally their diameter is larger than 10 mm) and this type of artery receives blood directly from the heart, so their walls contain high elastin concentrations, allowing them to stretch and support high blood pressure.
- Muscular arteries or distributing arteries (i.e., radial and femoral arteries), as the name indicates, distribute the blood to all the organs and tissues. This type of artery is composed of a high concentration of smooth muscles. The range diameter is 0.1 to 10 mm.
- Arterioles are the smallest type of artery. They work as resistors causing a drop in blood flow pressure and their average vessel diameter is 30 μm. The arteriole wall typically has one or two layers of smooth muscle.

**Group II: Veins and small branches (venules)**: the main function is taking the oxygen-poor and carbon dioxide-rich blood back to the heart. Typically, these vessels have low pressure and their classification is based on depth, function, and geometry. Similar to arteries, vein walls are constituted of three layers, however, they are much thinner. The diameter of veins is from 20 μm (venules) up to 30 mm for the vena cava (the major vein of the human body). Veins act as capacitors. Some veins have venous valves to prevent back-flow and to help maintain blood flow in the right direction to the heart. Usually, they are much more tortuous than arteries, precisely because of deformation resulting in high blood pressure and blood back-flow accommodating a large volume of blood before the venous valve [19,51].

**Group III: The capillaries**: capillaries have an important role in exchanging metabolic substrates and waste byproducts between blood and tissues. The capillaries make the connection between arteries and veins through arterioles and venules. They are narrow and thin-walled, making them the smallest of all the blood vessels (5–8 μm, inner diameter). Their walls consist of a single layer of endothelial cells, making the inner diameter just wide enough to allow red blood cells (erythrocytes) to pass through [19,51].

The dimensions and mechanical properties of the vessels are responsible for a non-negligible fluid–structure interaction (FSI) between the blood flow and the wall of the vessels. This topic has been recently reviewed by Campo-Deaño et al. [52] and it is not analyzed further in this work.

#### 4.1.2. Vascular Bifurcations

Blood vessels commonly bifurcate numerous times until they become capillaries. There are some sites where arteries can bifurcate, trifurcate, or even quadrifurcate [53,54,55]. A bifurcation consists of several geometric parameters such as bifurcation angle, planarity between all the branches, diameter ratio between the main branch and daughter branches, curvature, and length of the branches. The morphology of this type of blood vessel to ensure the best dynamic performance of a microbot is of paramount importance. In this location, the flow rate of the mother vessel is divided, and the velocity profile changes when it reaches the bifurcation apex. When blood flow enters the daughter branches, regions with flow separations appear and the flow behavior changes during the cardiac cycle.

### 4.2. Cardiac Cycle

The human heart could be defined as a simple and efficient flow pump whose operation (approximately 72 beats/min) is equivalent to 1.2 Hz. The duration of a beat is approximately 0.830 ms. The heart works as two pumps in series. To ensure this function, the blood travels unidirectionally through valves inside these pumps (heart valves), preventing the back-flow of blood. At the beginning of a heartbeat, a cardiac event correlates the blood flow and pressure that occurs until the next heartbeat. This event is of paramount importance and it is called the cardiac cycle. William Harvey was the first researcher demonstrating blood circulation under pulsatile flow where the amount of blood is finite [56]. The effect of changes in systole and diastole in blood pressure through the entire circulatory system took more than 100 years to be understood [51]. Diastole occurs when the heart muscle relaxes after contraction. Systole occurs when the heart muscle contracts to pump blood out from the heart. Moreover, a time-dependent function for blood pressure and flow rate regulation is the result of contraction and relaxation of the heart muscle during a heartbeat and it is traduced in a pressure wave (pulsatile flow) into the entire blood vessel network.

**Figure 5:**
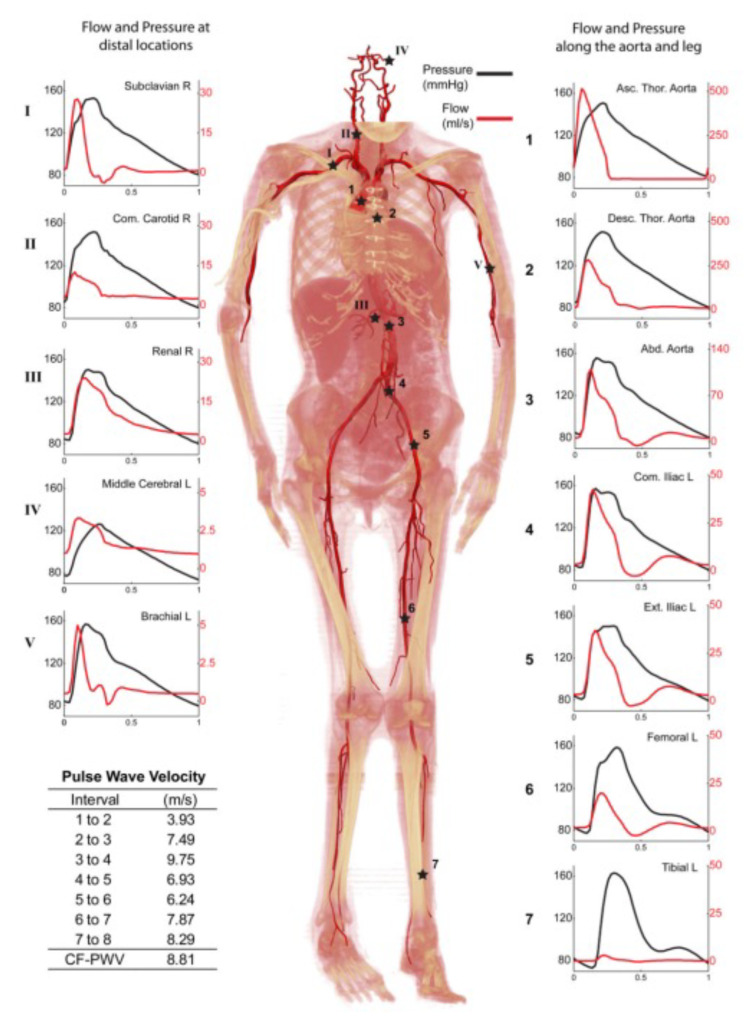
Pressure and flow waves at several sites in the body considered in the multi-stage computational model. Reprinted from Journal of Computational Physics, 244, Nan Xiao, Jay D. Humphrey, and C. Alberto Figueroa, Multi-scale computational model of three-dimensional hemodynamics within a deformable full-body arterial network, 22–40 (2013), with permission from Elsevier [58].

Unsteady, pulsatile flow through nearly all of the cardiovascular system dominates many aspects of the system. Pressure gradients regulating blood flow through the heart suffer a great variation as well as the constantly loaded and unloaded stresses applied to the artery wall [52,57]. Xiao and co-workers [58] investigated the arterial blood flow using a novel multi-stage computational fluid dynamics (CFD) analysis taking into account the deformability of the arterial network with FSI analysis. They extracted the pressure and flow waves at multiple sites of the arterial network model (Figure 5). Once more, it is evident that the unsteady feature of blood flow cannot be ignored when analyzing the blood flow surrounding a 3D microbot in a segment of a specific blood vessel.

Another important aspect of the circulatory system is the blood pressure along the full vasculature. Here, it is important to distinguish two types of circulation: macrocirculation and microcirculation. This classification could be carried out considering the network of large arteries and veins up to small arterioles and venules (macrocirculation). Here, the microcirculation starts, corresponding to the entire microvasculature involving the blood flow in arterioles, capillaries, and venules. Therefore, microcirculation is the last station to provide the nutrients for the living tissues of the system. Moreover, macrocirculation has a supporting function that consists of reducing the pressure oscillations resulting from the intermittent ventricular ejection and transforming the pulsatile flow into steady flow required for oxygen supply in the pulmonary microcirculation system [59]. However, the macrocirculation supporting function is entirely dependent on the viscoelastic properties of arterial walls and the vascular geometry, including diameter and length.

**Figure 6:**
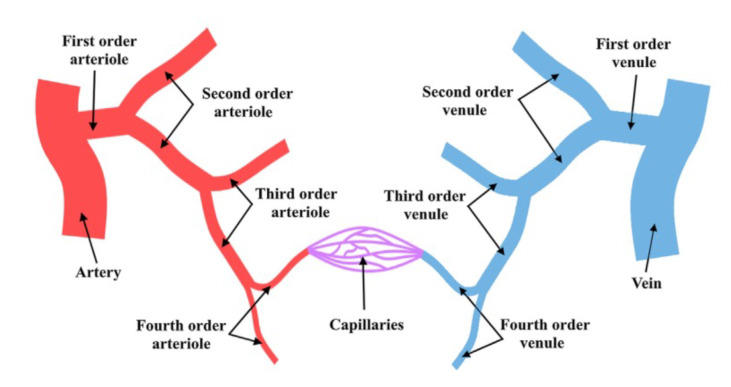
Simplified diagram of the microcirculation system. Reprinted from Biofluid Mechanics, Chapter 2, Ali Ostadfar, Microcirculation System (2016), with permission from Elsevier [57].

**Figure 7:**
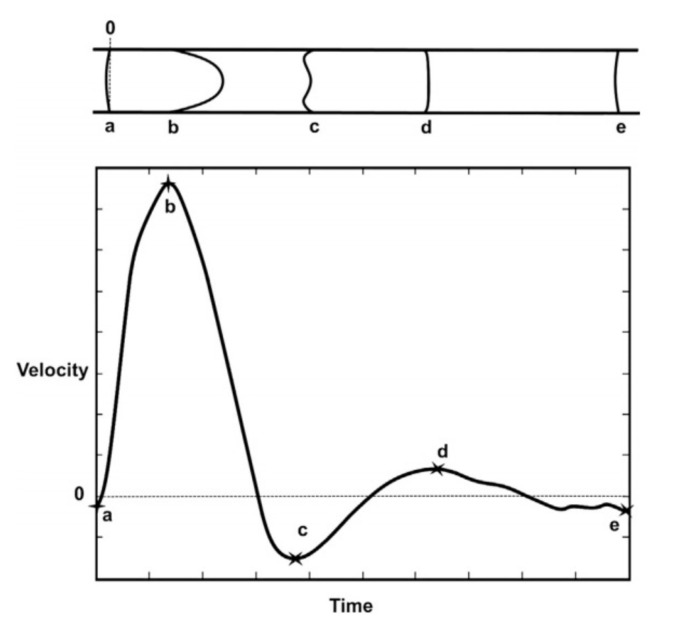
Flow velocity diagram, highlighting several points (a–e) during a cardiac cycle considering a vessel diameter of 5 mm (bottom) and respective velocity profile (top). Reprinted from Biofluid Mechanics, Chapter 2—Macrocirculation System, A. Ostadfar, 61–86 (2016), with permission from Academic Press [57].

Figure 6 shows a schematic representation of microcirculation. To better understand it, it is important to show the starting point which could be an artery or a vein and the subsequent subdivisions. Normally, this branching division could be categorized into four orders, with the fourth order being the connection to capillaries (the smallest vessels of the entire system) [60]. In this line, microcirculation is represented by blood vessels (arterioles, venules, and all the major capillaries) with a diameter range less than 150–180 μm. Here, a complex network of small vessels behaves with resistance in the entire vasculature in which nearly steady flow behavior is observed, as shown in Figure 7. High resistance is normally associated with the reduction in both pulsatile phenomena and steady flow, however, the diffusion of pulsatility through the entire vasculature should be the target of more study.

### 4.3. Characteristic Dimensionless Numbers

Taking into account the properties of human blood (density, viscosity, and relaxation time), the characteristics of the blood flow (velocity and frequency), and the dimensions of the main vessels (diameter), we define the set variables (k=6) and, considering that mass, length, and time (j=3) compound the independent dimensions appearing in these variables, the Buckingham Π theorem [61,62] states that just three dimensionless groups (n=k−j) are enough to characterize the hemodynamics. Thus, one should pick a set of three from the following dimensionless numbers:

**Reynolds number (Re)** defines the ratio of inertial forces to viscous forces when a fluid flows in a channel.

(1) $$\begin{array}{c} Re=\frac{\rho DV}{\mu } \end{array}$$

where ρ is the fluid density (kg/m${}^{3}$), μ is the dynamic viscosity (Pa·s), *D* is the diameter (m), and *V* is the fluid velocity (m/s). Normally, the Reynolds number is an asset to evaluate the fluid flow regime.

Table 2 shows the main features of main blood vessels in relation to their diameter, blood velocity, Reynolds number, wall thickness, and total blood volume. Typically, in the different blood vessels of the circulatory system, the blood flow is considered laminar (Re< 2000) only in the major arteries and veins could assume a transient behavior (Re> 2000). As we can see in Table 2, velocity becomes smaller with branching, and in capillaries the lowest value is 1 mm/s. All the blood vessels differ in dimensions, hemorheological behavior, and shear stress. Capillaries restrict robot size since their diameter is below 8μm. A rigid microbot larger than 5 μm in diameter would not succeed in capillary navigation. However, if the material is soft and highly deformable and similar to red blood cells, this function could be achieved, otherwise, accumulation or agglomeration could cause a stroke [63].

A wide range of complex fluids are characterized by their relaxation time (λ), and blood is not an exception. It is well documented that small length scales enhance the elastic forces, represented by the relaxation time, and they dominate over the viscous forces inducing elastic instabilities [64]. That is the reason why the relaxation of the blood, even when its value is small, may play an important role in the flow dynamics under pulsatile conditions. Associated with the relaxation time, two dimensionless numbers can be defined:

- **The Deborah number (De)** is the ratio of the time it takes the material to adjust to the applied stresses or deformation, i.e., the relaxation time and the characteristic time scale of the deformation process, i.e., the time of observation (*T*).
  (2) $$\begin{array}{c} De=\frac{\lambda }{T} \end{array}$$
- **The Weissenberg number (*****Wi*****)** is a dimensionless group representing the ratio between elastic forces and viscous forces [65].
  (3) $$\begin{array}{c} Wi=\frac{\lambda v}{D} \end{array}$$

Despite both numbers having different physical interpretations, they are wrongly used as synonyms [65].

**The Womersley number (α)** is the key factor for understanding and calculating the pulsatile flow and it is defined as the ratio of oscillatory inertia to viscous effects for a fluid in pulsatile flow:

(4) $$\begin{array}{c} \alpha =\frac{D}{2}\sqrt{\frac{\rho 2\pi f}{\mu }} \end{array}$$

where *f* is the fluid frequency (Hz), which can be seen as the reciprocal of the characteristic time of the deformation process defined above (*T*). In the circulatory system, the frequency generally is measured by the heartbeat rate. The Womersley number has an influence on the shape of the velocity profile and, typically for high values of α, an oscillating plug flow is observed, and it is evident that the flow is dominated by inertia. For small Womersley values, there is an equilibrium of viscous forces and the driving pressure gradient from the ratio of oscillatory inertia. Velocity waveforms or flow velocity waveform graphs are generally used for the cardiovascular system to better characterize the pulsatile flow. This implies the analysis of the flow velocity and its direction along the vessels. Figure 7 describes an example of a flow velocity waveform (bottom) and the respective velocity profile for a pulsatile flow along a random vessel. Several points (a–e) are compared, representing the velocity shapes of pulsatile blood flow velocity along a 5 mm diameter vessel. High peak velocities show a parabolic velocity profile and low peak velocities show more flattened velocity profiles.

Table 3 shows estimated values for the Womersley number in different sites of the vasculature [66] considering Newtonian blood flow behavior. However, in small vessels, the Non-Newtonian properties of blood should not be ignored, as discussed above, and it would be possible, considering a fully developed flow, to incorporate a more complex formula for the Womersley number.

**The Strouhal number** is a dimensionless number typically used in fluid mechanics to describe oscillating flows. The Strouhal number for a channel (or pipe) can be defined as:

(5) $$\begin{array}{c} St=\frac{fD}{V} \end{array}$$

This parameter is mostly considered in the study of unsteady oscillatory blood flow of arteries with irregular shapes and within aneurysm formation. Stalder et al. [67] investigated the aorta flow instabilities in thirty young healthy volunteers by flow-sensitive magnetic resonance imaging (MRI). For that purpose, they calculated and correlated the flow, Womersley, Strouhal, and critical Reynolds numbers. It is important to note that in the case of the blood flow through the aorta, the large value of the diameter combined with the low value of the relaxation time makes the Weissenberg number irrelevant. They observed the highest values of Re, α, and St in the ascending aorta and these parameters decreased in the descending aorta. Their analysis of in vivo data proves firstly that it is possible to correlate these hemodynamic parameters to assess flow instabilities in a segment of an artery and finally the data showed that the descending aorta might be more prone to flow instabilities than the aortic arch [67]. With this in mind, further in vitro and numerical studies should take these parameters into account in the analysis of microbot motion. Later, Mandal et al. [68] in their investigation explored the rheological parameters of blood flow in an artery model with an aneurysm by a hemodynamic numerical simulation concerning axisymmetric pulsatile flow over an aneurysm. Their results were focused on the effect of the hematocrit in a pulsatile flow and were shown by the analysis of wall shear stress (WSS) distribution, and the peak value of the WSS increases significantly with increasing St. This indicates once more that to evaluate the blood flow behavior around a microbot that will be designed for swimming in blood vessels, the pulsatile nature of blood flow, either experimentally or numerically, must be considered in order to obtain the best microbot dynamic performance and to minimize the effect of the flow disturbances in the normal circulation of blood.

Thus, for unsteady viscoelastic flows, as is the case of the flow of blood inside the human circulatory system, we can write

(6) $$\begin{array}{c} R=f(Re,Wi,\alpha ) \end{array}$$

considering that the Womersley number can be expressed as a combination of Re and $St=\frac{De}{Wi}$ and reduced to the following expression:

(7) $$\begin{array}{c} \alpha =\sqrt{\frac{\pi }{2}ReSt}=\sqrt{\frac{\pi ReDe}{2Wi}}. \end{array}$$

It is important to note that whenever there is more than one dimensionless group, dimensional analysis places no restriction on which groups we derive [65].

## 5. Pulsatile Flow

From all the information provided in Section 4, understanding the low Reynolds number pulsatile flow of viscoelastic fluids through small conducts is fundamental for the understanding of the blood flow in the human circulatory system.

The literature considering the unsteady flow of viscoelastic fluids in a channel is rather scarce, although it has been growing since the 1990s. In pulsating flows of viscoelastic liquids, the relation between dynamic and kinematic characteristics of the flow appears to be more complicated than in a Newtonian fluid, as the stress tensor is not related directly to the deformation rate tensor at any given instant, and the phase shift between the velocity and pressure gradient is not defined by flow inertia alone [69].

Several experimental studies have been developed at the macroscale, where the elastic forces are far less important than the viscous forces. They typically control the time-varying pressure gradient [69] or flow rate [70] and analyze the velocity profiles as a function of the pulsation phase and the pressure/flow profiles. With the advent of microfluidics, the number of experimental studies considering pulsating flows of viscoelastic flows has rocketed, due to the increasing number of applications that use this type of motion, such as chemical synthesis inside microfluidic channels, liquid–liquid extraction, mixing by oscillatory cross flow, cooling of microelectronic circuits by micro-oscillating heat pipes, inertial focusing of particles of a few microns, DNA elongation studies, or studies of the oscillatory movement of liquid plugs displaced by air in microchannels as model pulmonary flows [71]. It is also at the microscale where the elastic forces are enhanced due to the small scale [72].

Numerical studies of viscoelastic fluids in pulsating flows have also increased considerably in recent years, particularly because the instability or convergence issue at a high Weisenberg number (Wi) or Deborah number (De) has been successfully tackled with a variety of approaches that allow for stabilizing the numerical solution such as the log-conformation tensor approach, among others [73]. These techniques have allowed the development of numerical studies of the pulsatile flow of viscoelastic fluids in constricted stenosed arteries [74] and the analysis of the effect of body acceleration on the pulsatile flow of blood in a vessel [75].

Hydrodynamics around macroscopic objects, such as the canonical case of a cylinder in a cross-flow, have been thoroughly studied for Newtonian fluids for more than a century, since Prandtl’s conceptualization of the boundary layer [76]. However, the hydrodynamics for the case of unsteady cross-flow have been studied far less, with most studies dealing with the enhancement of heat transfer when compared to steady flows and, particularly, focusing on the lock-on effect (i.e., synchronization of vortex shedding frequency with the frequency of the forced pulsations) [77]. Regarding a numerical approach, the effects of pulsating blood flow on the dynamics of microbots have been recently analyzed by Ferreira and co-workers [78,79,80,81]; although they considered pulsatile blood velocity to determine the non-linear drag force over the microbot, they did not analyze the blood flow dynamics around the microbot. From the analytical perspective, Plotner et al. [82] investigated the magnetic propulsion of a microbot in pulsating flow; they concluded that gradient fields of around 200–400 mT/m could allow navigation in small blood vessels and reductions in the average and peak blood flow velocity are the key variables for practical use in a clinical environment. However, to the best of the authors’ knowledge, the hydrodynamics of the pulsating flow of viscoelastic fluids around objects have not been yet reported in the literature, neither experimentally nor numerically.

## 6. Final Remarks

Cardiovascular diseases, and particularly vascular obstructions due to thrombus formation of atheroma, are responsible for fatal accidents. There are different strategies to solve these health issues in order to prevent severe problems. Biomedical research aims to provide new solutions, which tend to be uninvasive as possible for the patient and this is where microbots may be of paramount importance for future treatments of CVDs. The most likely potential applications of the use of microbots in the human circulatory system have been reviewed, and the most recent and relevant microbot designs have been critically assessed.

The human circulatory system is a closed circuit consisting of a viscoelastic liquid, i.e., blood; channels with different dimensions and elasticity, which affect the flow dynamics of the blood, namely velocity and pressure; and a pump, i.e., the heart, which imposes a pulsating nature on the blood flow. This is a very complex environment that represents a true challenge for the navigation of medical microbots, from any perspective, i.e., analytical, numerical, or experimental. In this manuscript, we have also revised the state of the art of blood flow dynamics around medical microbots, highlighting the works that have been developed, their limitations, and the remaining empty gaps to be filled in this field of research. Regarding the design of the microbots, future works should try to reduce the size of the microbots to reach smaller vessels; moreover, studies on biocompatibility analysis of the microbots and long-term toxicity would allow for determining the most suitable materials to be used in the next generation of microbots for CVD treatments. Finally, the major challenge is determining how the pulsatile nature of blood flow may affect the performance of the microbots; the analysis of the blood flow dynamics around the microbots may provide relevant information for the definition of the right morphology of the microbots to obtain optimal performance. We hope this work motivates the community to work on the analysis of blood flow dynamics around microbots so that the generated knowledge may allow better control of microbot dynamics.

## Figures and Tables

**Table 1:** List of strategies for removal procedures of thrombi and atheroma according to Miloro’s classification [23] and where the new designs of microbots could be useful.

Procedure	Physical Mechanism	Microbot Design
Chemical	Chemical lysis	Ciliary microbot
		Soft attractor wall microbot
		Surface microroller
		Ciliary microbot
Chemomechanical	Chemical lysis + maceration	Self-folding microbot
		Helical microbot
Mechanical	Maceration	Sperm-shaped microbot
		Snake-shaped microbot
		Helical microbot
	Milling	Self-folding microbot
		Scallop-shaped microbot
		Helical microbot
	Dragging	Microrocket robot
Laser	Photoablation	Snake-shaped microbot

**Table 2:** Blood vessel characteristics, blood velocity, and estimated Reynolds number. Adapted from [60].

Blood Vessels	Inner Diameter	Blood Velocity	Reynolds Number
	[mm]	[mm/s]	[-]
Aorta	25	245–630	3000
Vena cava	30	50–300	3000
Arteries	2–6	100–500	110–850
Main artery branches	2–4	-	-
Terminal artery branches	1–2	-	-
Veins	5	3–50	150
Arterioles	0.03	1–100	0.7
Venules	0.02	<3	0.01
Capillaries	0.008	<1	0.002

**Table 3:** Womersley number estimation for different blood vessels considering a constant dynamic viscosity of 5×10${}^{-3}$ (Pa·s), a fluid density of 10${}^{-3}$ (kg/m${}^{3}$), and a fluid frequency of 1 (Hz). Adapted from [66].

Blood Vessels	Inner Diameter	α
Aorta	10	10
Large arteries	4	4
Small arteries	1	1
Arterioles	0.1	0.1
Capillaries	0.01	0.01
